# Milk, an Article of Food, Not a Mere Beverage

**Published:** 1856-01

**Authors:** 


*Milk, an Article of Food, not a mere Beverage.*—This has been determined legally the other day by the Court of Cassation in Paris, and its decision is in accordance with sound physiological principles. After this, the person who adulterates milk, no longer commits a simple contravention of the acts of police, with a penalty of 15 francs fine, and imprisonment of from 24 hours to 8 days; he now may be found guilty of a misdemeanor, and punished with a fine of 50 francs, and imprisonment of from three months to a year.—*Gazette des Hopitaux.*

